# Report on Epidemic Cholera

**Published:** 1867-09

**Authors:** 


Report on Epidemic Cholera. Circular No. 5. Surgeon Generals Office, War Department, May 4th, 1867.

This report is in pamphlet form, folio size, 65 pages; and contains a full account of the cholera as it affected the United States Army in 1866. It contains full statistical tables, and abstracts from official reports, of much value, in making up the statistics and history of this much dreaded disease.

